# Comparison of Specificity and Sensitivity of AMH and FSH in Diagnosis of Premature Ovarian Failure

**DOI:** 10.1155/2015/585604

**Published:** 2015-05-31

**Authors:** Farzaneh Alipour, Athar Rasekhjahromi, Mehrnoosh Maalhagh, Saeid Sobhanian, Masoumeh Hosseinpoor

**Affiliations:** ^1^Student Research Committee of Jahrom University of Medical Sciences, Jahrom, Iran; ^2^Jahrom University of Medical Sciences, Jahrom, Iran; ^3^Department of Community Health Nurse, Jahrom University of Medical Sciences, Jahrom, Iran

## Abstract

*Introduction*. Anti-Müllerian hormone represents the primitive follicular number and ovarian age. Low level of AMH is in relation to early menopausal state and decreased ovarian reserve. AMH level changes occur prior to FSH level in representing ovarian failure. The aim of this study is to compare sensitivity and specificity of AMH with FSH in diagnosis of POF. *Material and Methods*. This descriptive study is done on 96 patients referred to Dr. Rasekh Clinic. Serum level of AMH and FSH was measured at Day 3 (3rd day of menstrual cycle) and data were analyzed through SPSS 21 software. *Results*. Results of AMH and FSH serum level indicate that AMH has more sensitivity (80% versus 28.57%) and almost equal specificity (78.89% versus 78.65%) compared with FSH. Also negative predictive value of AMH (98.61%) and FSH (87.5%) is different. But positive predictive value is the same (17.39%). Diagnostic accuracy of AMH is more than FSH and has significant differences. *Conclusion*. According to the results of this study, AMH serum level is more sensitive than FSH serum level. Also AMH has more negative predictive value. Besides, this hormone can be measured at any time of menstrual cycle, against FSH. AMH seems to be more useful in early diagnosis of POF.

## 1. Introduction

Anti-Müllerian hormone (AMH) is a polypeptide, member of the transforming growth factor-*β* (TGF*β*) family. AMH gene is placed on chromosome 13 and its receptor is on chromosome 12 called AMHR2 [1, 2].

In healthy female fetus, this protein is detected in umbilical cord. Measurement of AMH in different stages of life shows that serum level of AMH decreases during the first four years and then it would increase linearly during the next four years and the serum level will not change during puberty and adolescence.

During reproductive age, AMH is secreted from granulosa layer cells of primary follicles which are developed from primordial follicles. AMH is secreted from small antral and preantral follicles which are less than 4 mm. Secretion of AMH reduces during follicle maturation and in follicles which are more than 8 mm AMH is not secreted anymore; therefore AMH serum level is constant during menstruation cycle [3]. On the other hand, maturation of follicles in each cycle is dependent on secretion of FSH which is different in phases of cycle, so basic level is measured on the third day of cycle [4].

The number of primordial follicles shows the ovarian age and storage. Computation of primordial follicles is impossible but follicles count during phase of development can lead to approximate number of primordial follicles and ovarian age [5]. According to the pattern of AMH secretion which is mostly in primordial follicles, serum level of AMH can show the age and storage of ovary [6].

Evaluation of ovarian storage is an essential step for treatment of infertility.

There are several methods for evaluation of ovarian storage such as antral follicular count (AFC) which is done by ultrasonography [7].

Other methods are measuring estrogen, FSH, and inhibin B serum levels with some restrictions such as time limitation for evaluation of the serum levels [7, 8].

Serum level of AMH is associated better with a decline of oocytes/follicles over time. AMH serum level can be measured at any time of cycle and the serum level is not affected by consumption of oral contraceptive pills (OCP), against serum level of FSH and other markers [9, 10].

Premature ovarian failure is one of the causes of infertility which is described as a decline in ovarian function and ovarian response to FSH and reduction of estrogen level [11, 12]. Incidence of POF and it occurs before forth decade of life earlier than physiologic menopause that occurs in fifth decade of life [13, 14].

Serum level of AMH decreases or cannot be detected in patients with POF (Table 1).

One of the benefits of AMH serum level measurement is to identify patients with risk factors of POF such as family history of POF, history of chemotherapy and radiation to pelvic region, and history of autoimmune diseases [15, 16]. Early detection of ovarian storage and numbers of primordial follicles decline can be used for prevention of infertility and preserving the remaining follicles [17].

Besides, patients with POF are prone to be involved with adverse effects of menopausal state; therefore early detection of POF can lead to primary prevention of these adverse effects such as osteoporosis and cardiovascular diseases [18].

The aim of this paper is to compare the sensitivity and specificity of AMH and FSH in detection of POF in patients with menstrual disorders diagnosed as primary ovarian failure in Jahrom city.

## 2. Material and Methods

### 2.1. Approval

The study was initiated after approval of Research and Ethics Committee of Jahrom University of Medical Sciences; after clarifying the study protocol, written consent was obtained from the participants.

### 2.2. Design

This is a descriptive study on 96 women who were referred to Dr. Rasekh Clinic due to menstrual disorders and history of infertility for one year.

Initially medical history and demographic information were assessed.

### 2.3. Hormone Measurements

Blood was collected on Day 3 (3rd of menstrual cycle), and serum was immediately separated by centrifugation for 6 min at room temperature. Serum AMH and FSH were measured by electrochemiluminescence immunoassay.

Exclusion criteria of the study are having history of autoimmune disease, radiation to pelvis, polycystic ovarian syndrome, and other medical or surgical disorders which are causes of menstrual disorders such as endometriosis, thyroid disease, and pelvic inflammatory disease. Inclusion criteria of the study are women with abnormal vaginal bleeding who are less than 40 years old, women who have decreased volume size of ovary or reduction of follicular numbers and size below normal range, and women with family history of premature ovarian failure:

 Normal size of ovary = 2.5–5 cm in length and 1.5 to 3 cm in width and depth.
 Volume of ovary (mL) = length (cm) × width (cm) × depth (cm) × 0.52.
 Normal count of ovary: 3–6 mm.

### 2.4. Statistical Analysis

Data were analyzed by SPSS for Windows (version 21.0.0; SPSS, Inc., Chicago, IL, USA). Then evaluation of binary classifier for determination of specificity and sensitivity was done.

## 3. Results

Serum level of AMH measured on Day 3 in women with abnormal vaginal bleeding for diagnosis of POF shows that sensitivity of this test in diagnosis of POF is 80% and specificity is 78.89%. Diagnostic accuracy of this test is 78.95%. Positive predictive value of AMH in diagnosis of POF is 17.39% and negative predictive value of AMH in diagnosis of POF is 98.61%.

Sensitivity of FSH in diagnosis of POF is 28.57% and specificity of FSH in diagnosis of POF is 78.65%. Diagnostic accuracy of this test is 71.84%.

**Figure 1:**
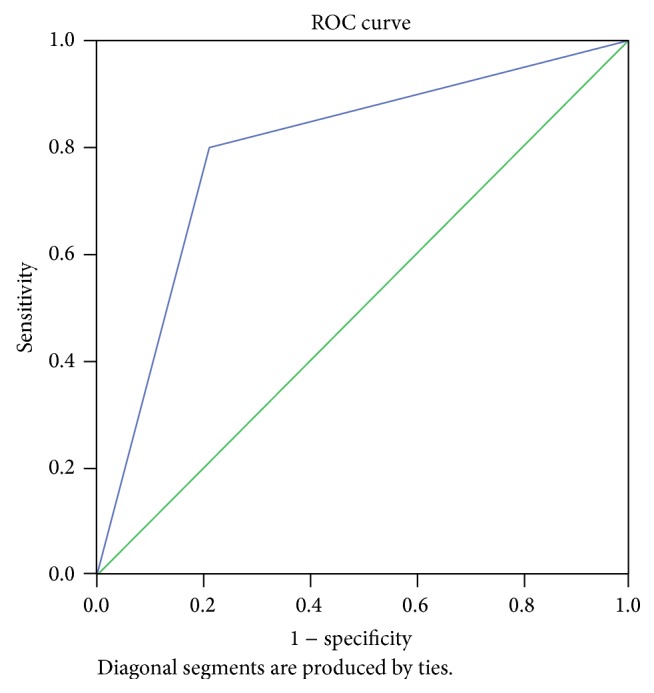


Positive predictive value of FSH in diagnosis of POF is 17.39% and negative predictive value of FSH in diagnosis of POF is 87.5% (Figure 1 and Table 2).

ROC curve shows that most of the cases that are diagnosed as POF also have positive serum level of AMH. ROC curve determines high sensitivity of serum level of AMH in diagnosis of POF (significance 0.027). Area in ROC curve shows that possibility of diagnosing POF with AMH serum level is 79.6%.

## 4. Discussion

Sensitivity of AMH in diagnosis of POF was more than that of FSH but both tests had almost equal specificity. Many scientists focused on relation of AMH level in diagnosis of POF but did not compare the specificity and sensitivity of AMH and FSH.

Diagnostic accuracy of AMH is more than FSH such that there is significant difference between the two tests (*P* value < 0.05). Therefore AMH is more reliable than FSH. Krawczuk-Rybak et al. showed that AMH is identified as ovarian function hormone in patients with lymphoma who have been treated by chemotherapy [19]. Also in different studies, AMH is a potential and reliable biochemical marker for predicting follicle quality in IVF and success in this procedure [20–22]. Dunlop and Anderson indicate in their study that AMH may also be a useful marker of cancer therapy-related ovarian damage in prepubertal children. AMH is proving to be of increasing value in assessing ovarian function [23]. The results of these studies are consistent with our findings. Miyazaki et al. clarified the importance of Days 1–5 E for predicting intermittent ovarian function [24]. As measuring AMH does not have these limitations it is a better method.

On the other hand, Dewailly et al. showed in their study that the value of AMH in the diagnosis of PCOS remains controversial, but it may replace AFC in the future [25]. This shows that scientists want to do more studies to confirm their hypothesis. Results of these studies and other similar studies concur that serum level of AMH shows ovarian storage and a valuable and reliable test in infertility workup.

## 5. Conclusion

Early diagnosis of reduction of ovarian storage can prevent expensive ways in treatment of infertile woman and in women who are susceptible to POF. As sensitivity of AMH in diagnosis of POF is more than FSH and specificity is equal, AMH is a better marker of POF but both tests can confirm each other having more reliable prediction. Also, oocytes freezing, ovarian tissue freezing, and embryo freezing are recommended for those who are at risk of POF.

## Figures and Tables

**Table 1:** AMH blood level.

AMH blood level	Interpretation
Over 3.0 ng/mL	High (often PCOS)
Over 1.0 ng/mL	Normal
0.7–0.9 ng/mL	Low normal range
0.3–0.6 ng/mL	Low
Less than 0.3 ng/mL	Very low

**Table 2:** Area under the curve.

Area	Std. error	Asymptotic sig.	Asymptotic 95% confidence interval	
			Lower bound	Upper bound
0.796	0.107	0.027	0.586	1.005
